# P-1441. Demographic and Clinical Characteristics of New Migrant Patients with HIV in a New York City Open Access HIV Primary Care Clinic

**DOI:** 10.1093/ofid/ofae631.1614

**Published:** 2025-01-29

**Authors:** Melissa Parkinson, Maureen Saylor, Jason Villarreal, Shaoli Chaudhuri, Shauna Gunaratne, Susan Olender

**Affiliations:** Columbia University, New York, New York; New York Presbyterian Comprehensive Health Program, New York, New York; New York Presbyterian Comprehensive Health Program, New York, New York; Columbia University, New York, New York; Columbia University Irving Medical Center, New York, New York; Columbia University Irving Medical Center, New York, New York

## Abstract

**Background:**

Since 2022, New York City has seen an influx of migrants, many seeking asylum due to political strife or discrimination. This study assessed the clinical characteristics of new migrant patients with HIV who sought care at an academic, open access clinic.

**Figure:**
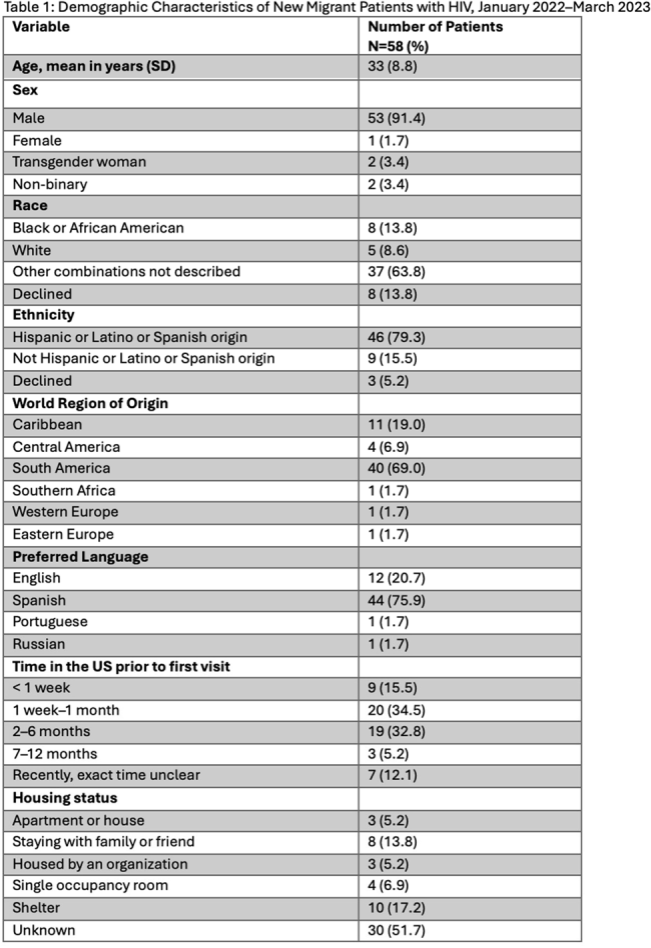

**Methods:**

This is a retrospective chart review study of new clinic patients January 2022–March 2023. Patients were included in the study if they were ≥18 years of age, living with HIV, and had migrated to the United States within one year of first clinic visit.

**Figure:**
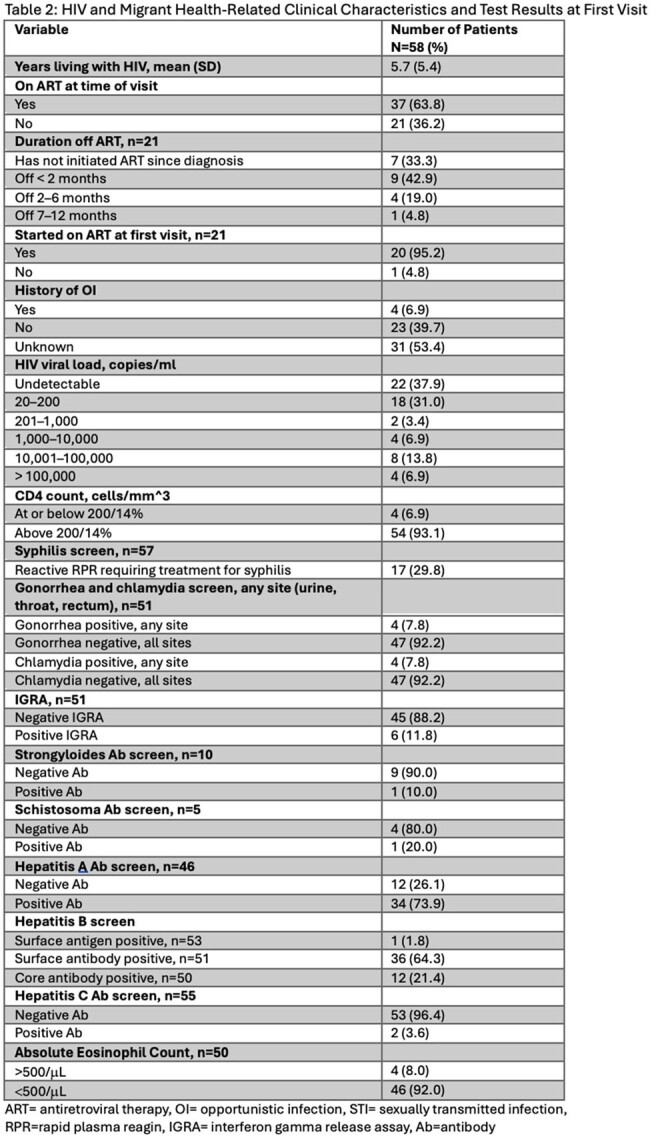

**Results:**

From January 2022 through March 2023, we identified 58 patients who met study criteria. 53 patients (91.4%) identified as male, and the mean age was 33 years (SD 8.8), see Table 1. Most patients were from South America, with just over half from Venezuela alone. 48.3% of patients were walk-ins and 43.1% were connected through an open access line, with a majority referred from community-based organizations. 37 patients (63.8%) were on ART (antiretroviral therapy) at first visit, see Table 2. 67% of patients had an HIV viral load of 200 copies/ml or less. 29.8% of patients had a reactive RPR (rapid plasma reagin) consistent with a current diagnosis of syphilis. 11.8% of patients screened had a positive IGRA (interferon gamma release assay), none with active tuberculosis. 17.9% and 10% of eligible patients were screened for Strongyloides and Schistosoma antibodies, respectively. Only 4 of 56 eligible patients received empiric treatment for helminthic infection. 46.6% of patients reported history of STI (sexually transmitted infection), see Table 3. Nearly all patients (93.1%) were linked to a care coordinator prior to or at first visit. 25 patients were identified as having a possible mental health disorder or history of trauma, and 44% were seen by a licensed social worker same day, see Figure 1.

**Figure:**
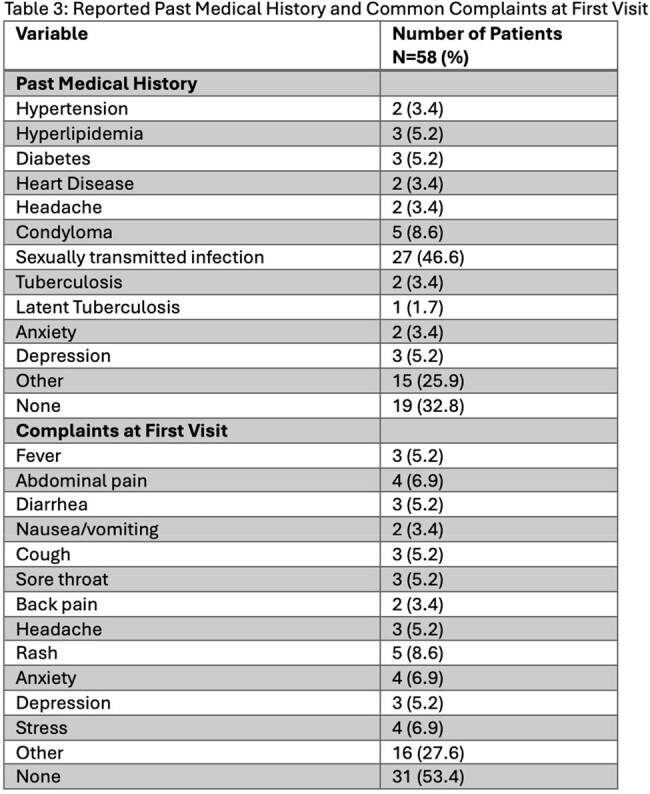

**Conclusion:**

Most new migrant patients were from South America, identified as male, and had suppressed HIV. There were missed opportunities to screen for and treat helminthic infections. There was a high burden of STIs and mental health disorders. Many patients utilized our open access line or walked into clinic and nearly all were connected to a care coordinator at or prior to first visit. Continued research is needed to better understand the needs of this vulnerable and growing patient population.

**Figure:**
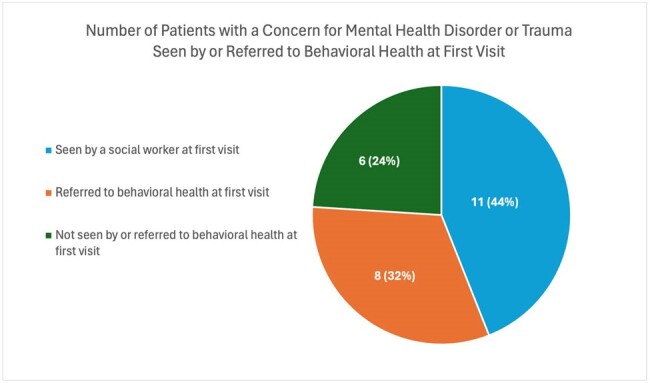

**Disclosures:**

**All Authors**: No reported disclosures

